# Impact of the VTE-PREDICT calculator on clinicians’ decision making in fictional patients with venous thromboembolism: a randomized controlled trial

**DOI:** 10.1016/j.rpth.2024.102569

**Published:** 2024-09-11

**Authors:** Daniël Duijzer, Maria A. de Winter, Marc Carrier, Alexander T. Cohen, John-Bjarne Hansen, Karin A.H. Kaasjager, Ajay K. Kakkar, Saskia Middeldorp, Henrik T. Sørensen, Frank L.J. Visseren, Philip S. Wells, Jannick A.N. Dorresteijn, Mathilde Nijkeuter

**Affiliations:** 1 Department of Acute Internal Medicine, University Medical Center Utrecht, Utrecht, the Netherlands; 2 Department of Internal Medicine, Diakonessenhuis Utrecht, Utrecht, the Netherlands; 3 Department of Medicine, University of Ottawa and the Ottawa Hospital Research Institute, Ottawa, Ontario, Canada; 4 Department of Haematological Medicine, Guys and St Thomas’ Hospitals, King’s College London, London, United Kingdom; 5 Department of Clinical Medicine, Thrombosis Research Center (TREC), Universitetet i Tromsø - The Arctic University of Norway, Tromsø, Norway; 6 Thrombosis Research Institute London, London, United Kingdom; 7 Department of Internal Medicine, Radboud University Medical Center, Nijmegen, the Netherlands; 8 Department of Clinical Epidemiology, Aarhus University Hospital and Aarhus University, Aarhus, Denmark; 9 Department of Vascular Medicine, University Medical Center Utrecht, Utrecht, the Netherlands

**Keywords:** clinical decision-making, hemorrhage, personalized medicine, randomized controlled trial, venous thromboembolism

## Abstract

**Background:**

After 3 months of anticoagulation for venous thromboembolism (VTE), the decision needs to be made whether to stop anticoagulation or extend treatment indefinitely. The VTE-PREDICT calculator can be used to estimate individual risks of VTE recurrence and bleeding to guide this decision.

**Objectives:**

To evaluate the impact of predicted individual risks of recurrence and bleeding on clinicians’ decisions on anticoagulation duration and to assess usefulness of the VTE-PREDICT calculator.

**Methods:**

A randomized controlled trial and within-subject study was conducted among clinicians treating VTE patients. The clinicians were asked to complete an online survey containing 6 fictional case vignettes. Group A proposed anticoagulant duration for each case without additional information first and subsequently after seeing calculator-predicted risks (within-subject analysis). Group B was directly provided with calculator risks and proposed treatment duration for each case vignette (for comparison with group A results in a randomized controlled trial analysis). Then, group B received questions on usefulness and credibility of the calculator.

**Results:**

Forty-five clinicians were assigned to group A and 48 to B. Overall, group A did not propose different anticoagulation durations than group B. However, individual clinicians in group A changed proposed duration in 35% of the cases after seeing the calculator risks. The calculator was considered useful and credible by most clinicians.

**Conclusion:**

Overall, use of the VTE-PREDICT calculator did not affect proposed anticoagulation duration. However, individual clinicians frequently changed their proposed duration after using the calculator, especially for patients with high bleeding risk.

## Essentials

• The VTE-PREDICT calculator informs on recurrence and bleeding risk after venous thromboembolism.
• This study investigated the effect of VTE-PREDICT on treatment duration in fictional patients.
• Use of VTE-PREDICT did not result in a different proposed anticoagulation treatment duration.
• The calculator was deemed useful by clinicians treating patients with venous thromboembolism.

## 1 Introduction

In patients with venous thromboembolism (VTE), anticoagulation therapy is recommended for at least 3 months [[1], [2], [3], [4]]. After this primary treatment, the decision needs to be made whether to continue anticoagulation as secondary prophylaxis for recurrent VTE since anticoagulants effectively reduce VTE recurrence risk but come at the cost of an increased risk of clinically relevant bleeding [5]. The decision should be the result of a process of shared decision making between patient and clinician, including consideration of patient preferences and a trade-off between risk of VTE recurrence and bleeding [[1], [2], [3]].

Extension of anticoagulant treatment for a definite period is now regarded as obsolete since it has been found to postpone rather than prevent recurrent VTE [6]. Current guidelines advise to either stop or extend anticoagulation indefinitely [2,3]. Indefinite treatment comprises anticoagulant therapy without a predefined end date but with periodical reassessment of recurrence and bleeding risk [2].

To determine optimal treatment duration, accurate estimations of VTE recurrence and bleeding risk are essential. However, in practice, this is challenging because both risks are highly heterogeneous among patients. As existing risk scores for recurrent VTE and bleeding have methodological shortcomings and insufficient predictive performance, they are not recommended for routine use in clinical practice [1,7]. Without risk scores, individual clinicians may weigh risk factors differently, resulting in different risk estimates for patients with the same characteristics [[8], [9], [10]]. Inaccurate and inconsistent risk estimation hampers informed treatment decisions, presumably leading to unfavorable treatment outcomes.

The VTE-PREDICT risk score was developed to predict individual risks of recurrence and clinically relevant bleeding in patients with VTE using 14 readily available patient characteristics [11,12]. The tool can be used to estimate individual 1-year and 5-year risks with and without extended anticoagulation using different anticoagulant agents and can thus inform shared decision making in clinical practice. To facilitate clinical use, the risk score was implemented in an online calculator that is freely accessible worldwide via www.vtepredict.com.

Given the increasing availability of online tools, clinicians should be critical of their ability to provide added value in clinical practice. However, studies on the impact of clinical tools are scarce and the optimal study design is yet to be determined [13]. One study has investigated use of a risk calculator on surgical treatment decisions using case vignettes, ie, a brief written description of a fictional patient in a particular situation mimicking real-world patients [14]. Case vignette studies can be used to investigate clinical decision making without involving patients. They constitute an ideal combination of traditional surveys and experimental methods, making the design suitable for studying the value of decision support tools [15].

This study aims to evaluate the effect of VTE-PREDICT–derived risks of recurrent VTE and bleeding on clinical decision making regarding anticoagulation duration. The study focuses on anticoagulation duration in adult patients with VTE, without active cancer, after the initial anticoagulant treatment. In addition, it investigates perceived usefulness and potential barriers to the use of the VTE-PREDICT risk calculator.

## 2 Methods

### 2.1 Study design

A randomized controlled trial (RCT) and within-subject study consisting of 2 online surveys was conducted among clinicians. The surveys were constructed and distributed using SurveyMonkey [16]. They contained 6 unique fictional case vignettes meeting predefined requirements for case vignette studies [15,17]. Each case vignette described a scenario of a typical VTE patient in which there is clinical equipoise on whether to stop or extend anticoagulant treatment after the initial treatment of 3 to 6 months. All cases, therefore, include patient characteristics for which guidance on treatment duration is inconsistent with current guidelines [[1], [2], [3], [4],^18^].

Five clinicians tested the case vignettes for complexity of the decisions and their real-world resemblance and relevance. The vignettes were then revised accordingly. Detailed information on the development process is provided in Supplementary Figure S1, and the case vignettes are presented in Supplementary Text S1.

Participating clinicians were randomized to either group A or B (allocation ratio 1:1). Both groups were asked to propose an anticoagulation duration for each case vignette without taking patient preferences into account. Proposed anticoagulation duration was assessed using a multiple-choice question with the following answer options “stop anticoagulation,” “extend anticoagulation for definite period (e.g. 3 or 9 months),” “extend anticoagulation indefinitely,” or “I would propose another treatment.” Group A (control group) was asked to decide on anticoagulation duration just as they would do in daily practice. After reviewing the 6 cases, clinicians of group A were presented with the individual-predicted risks and then again asked to propose an anticoagulation duration for the same 6 cases. Participants were not able to return to previous pages to change given answers. Participants randomized to group A who reported that they used the VTE-PREDICT calculator as a tool to inform decisions about treatment duration were excluded from further analysis. Group B (intervention group) was directly visually presented with individual 5-year risks of recurrent VTE and clinically relevant bleeding with and without anticoagulation as predicted by the VTE-PREDICT risk score for case vignettes 1, 2, and 3. Subsequently, they were asked to use the online calculator in case vignettes 4, 5, and 6. Accurate calculator risks were shown in all cases as well to ensure that the proposed treatment duration was based on equal risks among all study participants. Subsequently, they were asked 5 questions regarding their perception of the calculator on a Likert scale and 3 open-ended questions to collect feedback for improving the calculator (Supplementary Text S2).

**Figure 1:**
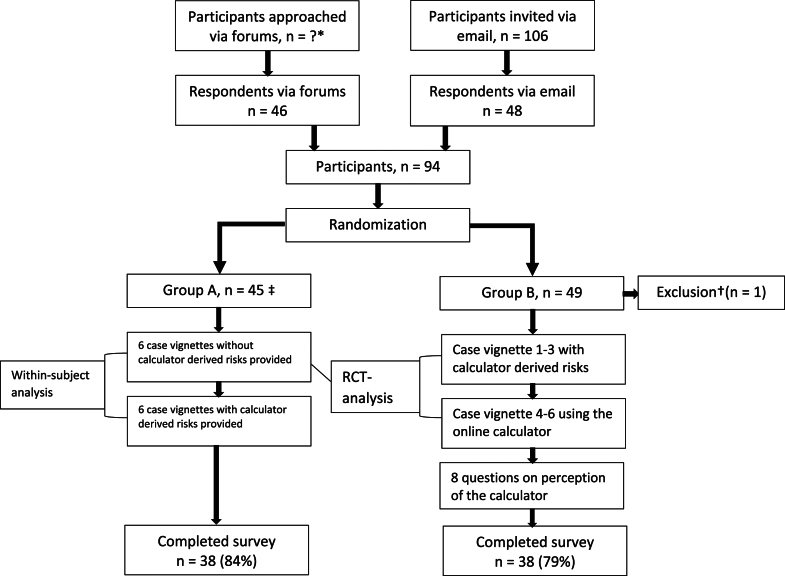
Flowchart: participants and surveys. RCT, randomized controlled trial. ∗Number of possible participants reached via the URL could not be determined with the method used. †Participant stated that she was a family nurse practitioner. ‡No participants randomized to group A reported that they used the VTE-PREDICT calculator as a tool to inform decisions about treatment duration in this study.

A flowchart of the study design is presented in Figure 1.

### 2.2 Participants

Medical specialists and residents treating VTE patients were eligible for participation in the study. They were recruited through the network of VTE-PREDICT advisory board members and through 2 online forums (International Society on Thrombosis and Haemostasis and ResearchGate) between December 1, 2022, and January 12, 2023. Randomization was carried out using a URL generated via www.nimblelinks.com, directing participants randomly to 1 of the 2 online surveys.

With an estimated 20% difference in the proposed anticoagulation duration between groups A and B, power of 80%, and α of 0.05, the minimal sample size was established on 28 participants based on results of 168 case vignettes. An inflation factor of 2.5 was taken into account, resulting in a final sample size of 70 participants [19]. The inflation factor was introduced to reduce the impact of intrarater correlation, as some participants may tend, in general, to prescribe extended anticoagulation more often. Without an inflation factor, this could have had a disproportional impact on the overall results.

### 2.3 Ethics

Due to its noninvasive design, this study was not subject to the Medical Research Involving Human Subjects Act. Participants were informed about the study’s aims; informed consent was obtained prior to participation, and no incentives were offered.

### 2.4 Outcomes

The outcomes of the RCT analysis were the proportions of clinicians stopping or extending anticoagulation treatment without and with use of the calculator-derived risks. For the within-subject analysis (group A), the outcomes were the proportions of changes in proposed treatment duration after being presented with the calculator-derived risks. For both analyses, the primary outcome was the sum of all case vignettes combined; individual case vignettes were analyzed as secondary outcomes.

A secondary outcome in both analyses was the consensus among clinicians in group A and group B regarding anticoagulation duration. Amount of consensus was defined as the proportion of the most frequent proposed treatment duration overall and for individual case vignettes. In addition, perceived usefulness, credibility, and intention to use were secondary outcomes queried in 5 5-point Likert scale questions (Supplementary Text S2).

### 2.5 Statistical analysis

Different proposed anticoagulation duration options (stopping or extending) were expressed as proportions per case vignette and overall mean proportions with 95% CIs. In the RCT analysis, proportions in groups A and B were compared using the chi-squared test. In the within-subject analysis in group A, the change in proposed anticoagulation duration with and without the use of predicted risks was assessed using Stuart–Maxwell’s test [20,21].

The options “stop anticoagulation” and “extend anticoagulation for definite period (e.g. 3 or 9 months)” were both considered treatments of limited duration and therefore analyzed jointly in the RCT analysis. Data from incomplete surveys were included in the final analysis when treatment duration was proposed in at least 1 case vignette. No imputation of missing data was performed.

All statistical analyses were conducted using IBM SPSS version 27. *P* values below .05 were considered statistically significant.

## 3 Results

A total of 94 clinicians took the survey, of which 45 were randomized to group A and 49 to group B. One participant in group B was a nurse practitioner and was therefore excluded, resulting in a total of 93 clinicians in the final analysis. Completion rates were 84% in group A and 79% in group B. Most clinicians were specialists (89%) in an area of internal medicine and affiliated with an academic hospital (75%; Figure 1; Table 1).

**Table 1:** Participant characteristics.

Characteristic	Group A (*n* = 45)	Group B (*n* = 48)
General information		
Sex (male)	32 (71)	26 (54)
Experience		
Estimated years treating VTE patients	15 (8-25)	11 (6-22)
Estimated no. of unique VTE patients per month	12 (5-30)	15 (7-40)
Medical specialty		
Hematology	21 (47)	20 (42)
Internal medicine	11 (24)	14 (29)
Vascular medicine	4 (9)	6 (13)
Pulmonology	3 (7)	3 (6)
Cardiology	1 (2)	1 (2)
Other^a^	5 (11)	4 (8)
Level of training		
Specialist	41 (91)	42 (88)
Resident	4 (9)	5 (10)
Other^b^	0 (0)	1 (2)
Type of healthcare facility		
Academic hospital	31 (69)	39 (81)
General hospital	12 (27)	8 (17)
Unknown	2 (4)	1 (2)
Region of residence		
Europe	29 (64)	26 (53)
North America	12 (27)	16 (33)
Other^c^	4 (9)	6 (12)
Contacted via		
URL on forums	23 (51)	25 (52)
Email	22 (49)	23 (48)
Completed survey	38 (84)	38 (79)

Data are given as median (IQR) or *n* (%).

VTE, venous thromboembolism.

a Group A: thrombosis (2), emergency medicine, rheumatology, and transfusion medicine; group B: clinical pharmacy, rheumatology, thrombosis, and vascular surgery.

b Researcher at university who is involved in treating VTE patients.

c Group A: Australia (2), Bolivia, and New Zealand; group B: Israel (2), Argentina, Australia, Kazakhstan, and Nigeria.

### 3.1 RCT analysis: group A (no risks provided) vs group B (risks provided)

Considering the results of all case vignettes, cessation of anticoagulants was proposed in 36% of the cases by clinicians assigned to group A and in 41% of the cases by clinicians assigned to group B. Indefinite extension of treatment was proposed in 56% of the cases before being provided with the calculator risks compared with 49% when provided with the risks. These differences were not statistically significant.

For the individual case vignettes, a significant difference in proposed anticoagulation duration emerged only in case vignette 5. The vignette described a woman with an unprovoked pulmonary embolism, her second VTE, who has a 5-year bleeding risk of 15% when the anticoagulants would be continued. Clinicians without access to predicted risks (group A) proposed a longer duration of anticoagulant treatment than clinicians who were provided with calculator-derived risks (group B) in this case vignette (Table 2).

**Table 2:** Results of randomized controlled trial analysis regarding duration of anticoagulant therapy: group A (no risks provided) vs group B (risks provided).

Treatment decision	Group A	Group B	*P* value
Total^a^	*n* = 261	*n* = 255	.21
Stop^b^	36% (30-42)	41% (35-47)	
Extend indefinitely	56% (50-62)	49% (42-55)	
Other	8% (5-12)	11% (7-15)	
Clinicians’ consensus^c^	76% (70-81)	67% (61-73)	
Case vignette 1	*n* = 45	*n* = 48	1.00
Stop	45 (100)	47 (98)	
Extend indefinitely	0 (0)	0 (0)	
Other	0 (0)	1 (2)	
Clinicians’ consensus	100%	98%	
Case vignette 2	*n* = 44	*n* = 47	.539
Stop	9 (21)	14 (30)	
Extend indefinitely	29 (66)	29 (62)	
Other	6 (14)	4 (9)	
Clinicians’ consensus	66%	62%	
Case vignette 3	*n* = 43	*n* = 45	1.00
Stop	9 (21)	10 (22)	
Extend indefinitely	33 (77)	34 (76)	
Other	1 (2)	1 (2)	
Clinicians’ consensus	77%	76%	
Case vignette 4	*n* = 43	*n* = 39	.356
Stop	23 (54)	16 (41)	
Extend indefinitely	17 (40)	17 (44)	
Other	3 (7)	6 (15)	
Clinicians’ consensus	54%	44%	
Case vignette 5	*n* = 43	*n* = 38	*.004*
Stop	3 (7)	10 (26)	
Extend indefinitely	33 (77)	16 (42)	
Other	7 (16)	12 (32)	
Clinicians’ consensus	77%	42%	
Case vignette 6	*n* = 43	*n* = 38	.529
Stop	4 (9)	7 (18)	
Extend indefinitely	35 (81)	28 (74)	
Other	4 (9)	3 (8)	
Clinicians’ consensus	81%	74%	

Data are given as % (95% CI) or *n* (%). *P* values were calculated using chi-squared tests*. Italics* indicate statistical significance. Consensus percentage refers to the proportion of clinicians choosing the most frequently proposed anticoagulation duration option per case vignette.

a Results of all case vignettes combined.

b Composite of answer options “stop anticoagulation” and “extend anticoagulation for definite period (e.g. 3 or 9 months)”; also applies to results of individual case vignettes.

c Proportion of the most frequently proposed treatment duration.

### 3.2 Within-subject analysis: group A (before being provided predicted risks) vs group A (after being provided predicted risks)

Overall, clinicians randomized to group A changed 35% (95% CI, 29%-42%) of their proposed treatment durations when provided with calculator-derived risks.

For the individual case vignettes, the proportion of changes varied substantially, ranging from 5% to 67%. A significant proportion of changes were made in case vignettes 2 and 5. Case vignette 2 described a woman with unprovoked first-time deep vein thrombosis with a 5-year bleeding risk of 22%. Most of the changes favored a shorter anticoagulation duration (vignette 2, 69%; vignette 5, 67%; Table 3; Supplementary Table S1).

**Table 3:** Results of the within-subject analysis: group A (no risks provided) vs group A (risks provided).

Case vignette	Complete cases, *n*	Change in proposed treatment duration		
		*n* (%)	Type^a^, %	*P* value
Total^b^	231	81 (35)		*.001*
95% CI		29%-42%		
Case vignette 1	40	2 (5)		.50
Case vignette 2	39	26 (67)		*<.001*
Case vignette 3	38	9 (24)		.22
Case vignette 4	38	16 (42)		.268
Case vignette 5	38	21 (55)		*.001*
Case vignette 6	38	6 (16)		.53
				

*P* values were calculated using the Stuart–Maxwell test for marginal homogeneity. *Italics* indicate statistical significance.

No changes.

Changes favor shorter anticoagulant duration.

Changes favor longer anticoagulant duration.

Includes “other” in the proposed treatment duration.

a Further specification of changes in proposed treatment duration can be found in Supplementary Table S1.

b Results of all case vignettes combined.

### 3.3 Consensus among clinicians

There was a wide range of consensus among clinicians concerning treatment duration in the different case vignettes. The consensus among clinicians assigned to group B was 9% lower overall and 2% to 35% lower in individual case vignettes compared with the consensus among clinicians assigned to group A. These differences did not reach statistical significance (Table 2).

### 3.4 Other proposed treatments

The answer option “I would propose another treatment” was chosen for a minority of the cases (group A [no risks provided], 8%; group A [after risks provided], 6%; group B, 10%). Participants explained that they 1) would want to obtain more information on risk factors not mentioned in the vignettes before proposing a treatment duration, 2) would propose an anticoagulant other than the one mentioned in the vignette, or 3) would propose a treatment from which no duration could be deduced. (Supplementary Table S2).

### 3.5 Assessment of usefulness and credibility

**Figure 2:**
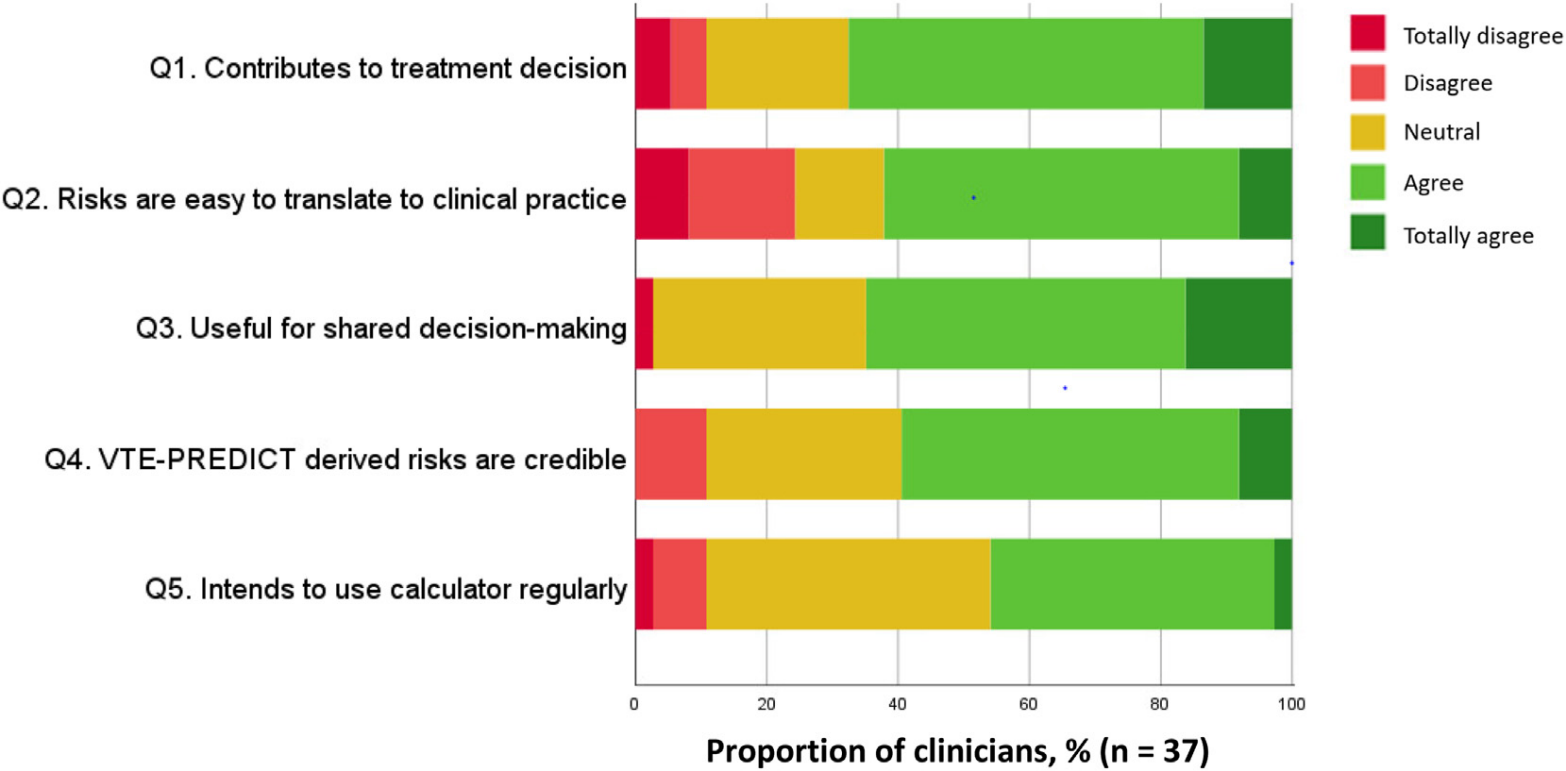
Assessment of the usefulness of, credibility of, and intention to use the VTE-PREDICT calculator (group B). The full text of questions on usefulness, credibility, and intention to use are provided in Supplementary Text S2.

Considering usefulness of the calculator, 68% of clinicians indicated that the calculator contributed to their treatment proposal in the case vignettes and 65% deemed it useful for shared decision making. Still, 24% of clinicians stated that they found it difficult to translate the presented risks to clinical practice. A minority (10%) questioned the credibility of the risks predicted with the calculator. Nearly half of the participants (46%) indicated that they intend to use the calculator regularly in clinical practice (Figure 2).

## 4 Discussion

This randomized controlled case vignette study shows that the VTE-PREDICT calculator influences clinical decision making on anticoagulation duration in specific VTE patients. On an individual level, providing risk predictions resulted in clinicians changing their decisions in over one-third of the presented cases. In addition, the risk calculator was considered useful by clinicians treating VTE patients.

Since the individual case vignettes differ greatly from one another, they need to be evaluated separately to draw conclusions.

Case vignettes 2 and 5 were the only vignettes in which a statistically significant effect of the risk calculator was observed (vignette 2, within-subject analysis; vignette 5, RCT and within-subject analysis). Case vignette 2 described an 85-year-old female with a first unprovoked proximal deep vein thrombosis, and case vignette 5 concerned a 72-year-old female with an unprovoked pulmonary embolism. Interestingly, both patients had a high 5-year bleeding risk when on anticoagulants—22% and 15%, respectively—compared with the bleeding risks of patients in the other vignettes (all below 10%). In both cases, the differences (RCT analysis) and changes (within-subject analysis) in proposed treatment duration when using VTE-PREDICT mainly favored a shorter anticoagulation duration. This suggests that the VTE-PREDICT calculator may have a more pronounced impact on treatment decisions for patients at high risk of bleeding. There are several possible reasons for this observation.

First, traditionally, clinicians often consider major bleeding only when assessing a patient’s bleeding risk. In contrast, VTE-PREDICT predicts a combined major and clinically relevant, nonmajor bleeding risk. The calculator-predicted risks are therefore higher than the risk typically taken into account by many clinicians.

Second, previous studies have shown that when deciding to stop or continue anticoagulation after VTE, clinicians and patients tend to focus on reducing the VTE recurrence risk rather than the increased bleeding risk associated with continued anticoagulant use [9,22,23]. The presentation of bleeding risks provided by the calculator vividly indicates the disadvantages of indefinite anticoagulation. For example, the within-subject analysis of case vignette 2 showed a significant number of treatment changes (67%), but the RCT analysis yielded no significant difference in the proposed treatment duration. Additional analyses showed that the proposed treatment durations of group A after being provided with the risks were significantly different (*P* = .001) from the proposed treatment durations of group B for case vignette 2. As these decisions were made with the same information available, the findings may be related to the sequential study design for group A. Clinicians in group A may have been surprised after seeing the patient’s high 5-year bleeding risk on anticoagulants (22%). On a group level, they might have overcompensated for their own lower risk estimate by frequently proposing shortening of anticoagulant treatment (69% of the made changes).

The most remarkable result of case vignette 4 was the low consensus among clinicians (group A, 54%; group B, 44%). This case vignette included a patient using oral contraceptives for 10 years prior to the event, a factor that is a subject of debate considering the extent to which it actually provokes VTE [24,25]. The calculator-derived risks led to a change in the proposed treatment duration among 42% of the clinicians in group A, with no clear preference for shortening or extending duration of treatment. In addition, the answer “I would propose another treatment” was selected more frequently in this case vignette than in the others. These results possibly reflect uncertainty about the extent to which the VTE should be considered provoked. An effect of the risk calculator on proposed treatment duration was not found in the analyses.

For case vignettes 1, 3, and 6, proposed treatment duration was the same with and without use of the calculator-derived risks. The high amount of consensus on treatment duration among clinicians may be related to the clearly provoked VTE, justifying short-term anticoagulation only (case vignette 1), and recurrent high-risk pulmonary embolism and unprovoked pulmonary embolism, making indefinite anticoagulation the most preferred treatment strategy (case vignettes 3 and 6, respectively) [10].

Another possible explanation is that the individual risks predicted by clinicians corresponded well with the risks predicted by the calculator. This would of course preclude changing treatment duration based on the calculator-derived risks.

Remarkably, the consensus on treatment duration was lower in all case vignettes after using the risk calculator. One reason may be that individual risks were weighed differently among clinicians. The VTE-PREDICT calculator presents recurrence and bleeding risks as absolute numbers as well as visually using 2 bar diagrams. However, the calculator does not provide advice on how to weigh these risks. Many clinicians may instinctively weigh risks in a 1:1 ratio, ie, consider that a bleeding event is as severe as a recurrent VTE event. Others will place more emphasis on either recurrent VTE or bleeding. In reality, the burden of VTE recurrence and bleeding—taking into account morbidity, mortality, side effects of treatment, and patient preference—may differ from one another. The proposed anticoagulation duration depends largely on how a clinician weighs the impact of the separate events.

Another explanation may be that individual clinicians in group B relied to a varying extent on the calculator-predicted risks, as most are accustomed to proposing a treatment duration based on general recurrence risks rather than individual risks. Besides, 10% of the participants disagreed with the statement that “the absolute risks derived from the VTE-PREDICT calculator are credible” (Figure 2). Therefore, presumably, some clinicians of group B did not base their treatment decision on calculator-predicted risks but rather on other factors, leading to proposed treatment durations that differed from those suggested by their colleagues who used the individual-predicted risks.

It should be highlighted that a small or even absent effect of the VTE-PREDICT calculator on clinicians’ decision making does not necessarily impair its utility. In general, clinicians had a positive attitude toward the calculator and considered it a useful tool. A vast majority stated that the calculator contributed to their treatment decisions, regardless of whether they made changes. This indicates that use of the risk calculator, even in cases with no effect on clinical decision making, strengthens clinicians in their treatment decisions. In addition, most clinicians deemed the calculator useful in shared decision making, as shown in a typical reflection by a participant, who stated that use of VTE-PREDICT “makes your treatment policy more transparent, both for the patient and the doctor.”

This study was the first to evaluate the effect of a risk calculator on clinical decision making for patients with VTE. This was assessed using 6 very different clinical case vignettes, mimicking VTE patients encountered in clinical practice, and clinicians from many countries participated [18]. As the calculator is available online (www.vtepredict.com), the results of this study can be applied to many VTE patients worldwide. However, several limitations must be addressed.

First, only a limited number of case vignettes could be included in the surveys, so the cases represent a selected sample of the range of VTE patients. Choosing other risk factors in the case vignettes could have led to substantially different results. However, by demonstrating an effect of the calculator on clinical decision making in some case vignettes, it can be assumed that calculator-derived risks would also influence clinical decision making in daily practice.

Second, patients’ preferences were not provided in the case vignettes, while this should be a major factor when deciding on anticoagulant treatment duration. In addition, social, financial, and legal considerations were not included in the cases. These factors may also play an important role in treatment decisions in clinical practice.

Third, although the participants consisted of the intended end-users of the VTE-PREDICT calculator, they might not constitute a representative sample. Most participants were specialists affiliated to an academic hospital, while the calculator is targeted to all caregivers treating VTE patients. Besides, many participants had extensive experience in treating VTE patients. If a group of clinicians with less experience was chosen, the calculator might have had a larger effect.

Fourth, while clinicians from many countries worldwide were included, it is known that preferences of clinicians on anticoagulant regimens differ geographically [26]. This may have introduced some variation in the results of the RCT analysis.

## 5 Conclusion

On a group level, the VTE-PREDICT risk calculator did not affect clinicians’ proposed duration of anticoagulation treatment in VTE patients. However, individual clinicians revised their decision concerning treatment duration after being shown VTE-PREDICT–derived risks in over a third of the cases. Especially in patients with high predicted bleeding risk, treatment was discontinued more frequently when using the calculator. Finally, clinicians deemed the VTE-PREDICT calculator useful for treating VTE patients.

## References

[1] Ortel T.L., Neumann I., Ageno W., Beyth R., Clark N.P., Cuker A. (2020). American Society of Hematology 2020 guidelines for management of venous thromboembolism: treatment of deep vein thrombosis and pulmonary embolism. Blood Adv.

[2] Stevens S.M., Woller S.C., Kreuziger L.B., Bounameaux H., Doerschug K., Geersing G.J. (2021). Antithrombotic therapy for VTE disease: second update of the CHEST guideline and expert panel report. Chest.

[3] Konstantinides S.V., Meyer G., Becattini C., Bueno H., Geersing G.J., Harjola V.P. (2020). 2019 ESC guidelines for the diagnosis and management of acute pulmonary embolism developed in collaboration with the European Respiratory Society (ERS). Eur Heart J.

[4] Kakkos S.K., Gohel M., Baekgaard N., Bauersachs R., Bellmunt-Montoya S., Black S.A. (2021). Editor’s choice – European Society for Vascular Surgery (ESVS) 2021 clinical practice guidelines on the management of venous thrombosis. Eur J Vasc Endovasc Surg.

[5] Alexander P., Visagan S., Issa R., Gorantla V.R., Thomas S.E. (2021). Current trends in the duration of anticoagulant therapy for venous thromboembolism: a systematic review. Cureus.

[6] Boutitie F., Pinede L., Schulman S., Agnelli G., Raskob G., Julian J. (2011). Influence of preceding length of anticoagulant treatment and initial presentation of venous thromboembolism on risk of recurrence after stopping treatment: analysis of individual participants’ data from seven trials. BMJ.

[7] de Winter M.A., van Es N., Büller H.R., Visseren F.L.J., Nijkeuter M. (2021). Prediction models for recurrence and bleeding in patients with venous thromboembolism: a systematic review and critical appraisal. Thromb Res.

[8] Ten Cate V., Prins M.H. (2017). Secondary prophylaxis decision-making in venous thromboembolism: interviews on clinical practice in thirteen countries. Res Pract Thromb Haemost.

[9] de Winter M.A., Remme G.C.P., Kaasjager K.H.A.H., Nijkeuter M. (2019). Short-term versus extended anticoagulant treatment for unprovoked venous thromboembolism: a survey on guideline adherence and physicians’ considerations. Thromb Res.

[10] Cate V.T., Lensing A.W.A., Weitz J.I., Beyer-Westendorf J., Wells P.S., Mismetti P. (2019). Extended anticoagulant therapy in venous thromboembolism: a balanced, fractional factorial, clinical vignette-based study. Haematologica.

[11] de Winter M.A., Büller H.R., Carrier M., Cohen A.T., Hansen J.B., Kaasjager K.A.H. (2023). Recurrent venous thromboembolism and bleeding with extended anticoagulation: the VTE-PREDICT risk score. Eur Heart J.

[12] Kaatz S., Ahmad D., Spyropoulos A.C., Schulman S., Subcommittee on Control of Anticoagulation (2015). Definition of clinically relevant non-major bleeding in studies of anticoagulants in atrial fibrillation and venous thromboembolic disease in non-surgical patients: communication from the SSC of the ISTH. J Thromb Haemost.

[13] Mansmann U., Rieger A., Strahwald B., Crispin A. (2016). Risk calculators—methods, development, implementation, and validation. Int J Colorectal Dis.

[14] Sacks G.D., Dawes A.J., Ettner S.L., Brook R.H., Fox C.R., Russell M.M. (2016). Impact of a risk calculator on risk perception and surgical decision making: a randomized trial. Ann Surg.

[15] Evans S.C., Roberts M.C., Keeley J.W., Blossom J.B., Amaro C.M., Garcia A.M. (2015). Vignette methodologies for studying clinicians’ decision-making: validity, utility, and application in ICD-11 field studies. Int J Clin Heal Psychol.

[16] Momentive Inc (2023). SurveyMonkey. http://www.surveymonkey.com.

[17] Sheringham J., Kuhn I., Burt J. (2021). The use of experimental vignette studies to identify drivers of variations in the delivery of health care: a scoping review. BMC Med Res Methodol.

[18] de Winter M.A., Uijl A., Büller H.R., Carrier M., Cohen A.T., Hansen J.B. (2023). Redefining clinical venous thromboembolism phenotypes: a novel approach using latent class analysis. J Thromb Haemost.

[19] McCarthy W. (2007).

[20] Stuart A. (1955). A test for homogeneity of the marginal distributions in a two-way classification. Biometrika.

[21] Maxwell A.E. (1970). Comparing the classification of subjects by two independent judges. Br J Psychiatry.

[22] van de Brug A., de Winter M.A., Ten Wolde M., Kaasjager K., Nijkeuter M. (2022). Deciding on treatment duration for unprovoked venous thromboembolism: what is important to patients?. Thromb Haemost.

[23] Lutsey P.L., Horvath K.J., Fullam L., Moll S., Rooney M.R., Cushman M. (2018). Anticoagulant preferences and concerns among venous thromboembolism patients. Thromb Haemost.

[24] Middeldorp S., Iorio A. (2017). Oral contraceptive use is a provoking factor for venous thromboembolism. BMJ.

[25] Rodger M.A., Le Gal G., Anderson D.R., Schmidt J., Pernod G., Kahn S.R. (2017). Validating the HERDOO2 rule to guide treatment duration for women with unprovoked venous thrombosis: multinational prospective cohort management study. BMJ.

[26] Palareti G., Bignamini A.A., Cini M., Li Y.J., Urbanek T., Madaric J. (2021). Anticoagulation duration after first venous thromboembolism: real-life data from the international, observational WHITE study. Clin Appl Thromb Hemost.

