# Supplementary material for: Impact of the VTE-PREDICT calculator on clinicians’ decision making in fictional patients with venous thromboembolism: a randomized controlled trial
Source: Res Pract Thromb Haemost. 2024 Sep 11;8(7):102569. doi: 10.1016/j.rpth.2024.102569 (PMC11491954; doi:10.1016/j.rpth.2024.102569)
Supplement: Supplementary Material [file mmc1.docx]

# Supplements

## Supplementary Figure S1. Step-by-step plan case vignette development.

**Step 1:** Select 1 cluster of typical VTE patient*


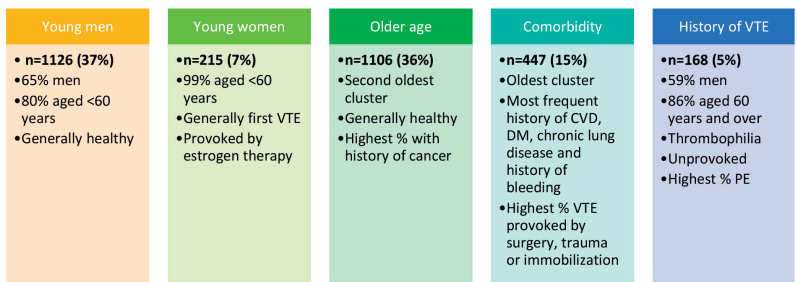


**Step 2:** Ensure patient meets the following criteria

- Aged >18 years
- First or recurrent lower extremity DVT and/or pulmonary embolism
- Using anticoagulation for 3 to 12 months after VTE
- No active cancer

**Step 3:** Selection of predefined clinical characteristics

- Presence or absence of provoking factors for VTE
- Relevant medical history and substance abuse
- Relevant medication use (e.g. antiplatelet therapy)
- Relevant clinical parameters (e.g. blood pressure)

**Step 4:** Selection of treatment

Initial treatment

- Apixaban (full dose)
- Rivaroxaban (full dose)
- Vitamin K antagonist with low molecular weight heparin (stopped after adequate INR)

Proposed extended treatment

- Apixaban (reduced dose)
- Rivaroxaban (reduced dose)
- Vitamin K antagonist

**Step 5:** Compose case vignette with selected variables and check whether

guidelines are inconsistent on treatment advice

**Step 6:** Repeat step 1 to 4 eight times independently

**Step 7:** Check whether frequency of separate characteristics from

the predefined extensive list is distributed reasonably among

the nine case vignettes

**Step 8:** Pre-testing of case vignettes: one clinician was asked to answer

the following questions for all nine case vignettes:

- Is the patient in this case representative for a real patient in

clinical practice?

- Does this case raise doubt on whether you would continue or

stop anticoagulation?

- Does this case lack essential information which would

influence your treatment proposal?

- Are there any uncertainties or ambiguities in this case?
- Do you have other comments on this case?

**Step 9:** Revision of case vignettes on clinical resemblance, doubt on optimal

treatment duration and clarity

**Step 10:** Repeat step 8 and 9 four times independently

**Step 11:** Selection of the 6 most suitable case vignettes out of the 9

developed

*Patient clusters replicated with permission from De Winter et al [18].

## Supplementary Text S1.1. Included case vignettes.

NB. guideline recommendations as summarized below each case vignette were not shown to participants during the survey.

*Case vignette 1*

The patient is a 52-year-old male who broke his right ankle in a running accident 5 months ago. His right lower leg was immobilized by a plaster cast subsequently. At a routine follow-up 1 month later, he complained of a swollen and painful right leg which was diagnosed as a proximal DVT. A vitamin K antagonist and a LMWH were started; the latter being stopped after 12 days.

Medical history shows no prior VTE and he is otherwise healthy. He consumes 2 alcoholic beverages per day and the family history shows that his mother had had a provoked deep venous thrombosis with pulmonary embolism at approximately the same age.

The ankle fracture has healed completely and he has been using the vitamin K antagonist for 4 months now. His INR values have been slightly above target range multiple times without any bleedings. Today you will propose whether to stop or continue the vitamin K antagonist.

|  | | **5-year risk** |  |  |
| --- | --- | --- | --- | --- |
| **Without anticoagulation** | | |  |  |
| VTE recurrence | | 8.9% |  |  |
| Clinically relevant bleeding | | 1.9% |  |  |
| **With vitamin K antagonist** | | |  |  |
| VTE recurrence | | 1.4% |  |  |
| Clinically relevant bleeding | | 9.8% |  |  |
|  | |  | | |
| **Guideline** | **Summary recommendation** | | | |
| ASH 2020[1] | Typically stopping anticoagulation | | | |
| CHEST 2021[2] | Suggestion of stopping anticoagulation | | | |
| ESC 2019[3] | NA | | | |
| ESVS 2020[4] | Considering stopping anticoagulation | | | |
|  | | | |  |

*Case vignette 2*

The patient is an 85-year-old female who visited the emergency department because of a first unprovoked proximal DVT of her right leg 4 months ago. Rivaroxaban (DOAC) was started upon diagnosis.

Her medical history comprises a hip fracture after a fall at home 1 year ago and breast cancer, which was considered cured after mastectomy, 20 years ago. She does not use any relevant medicine.

She has been treated with full dose rivaroxaban for 4 months now. Today you will propose whether to continue with rivaroxaban in a reduced dose or stop it.

|  | | **5-year risk** |  |
| --- | --- | --- | --- |
| **Without anticoagulation** | | |  |
| VTE recurrence | | 14.5% |  |
| Clinically relevant bleeding | | 6.3% |  |
| **With rivaroxaban (reduced)** | | |  |
| VTE recurrence | | 2.0% |  |
| Clinically relevant bleeding | | 21.8% |  |
|  | |  | |
| **Guideline** | **Summary recommendation** | | |
| ASH 2020[1] | Unclear | | |
| CHEST 2021[2] | Recommends indefinite anticoagulation | | |
| ESC 2019[3] | NA | | |
| ESVS 2020[4] | Recommends indefinite anticoagulation | | |

*Case vignette 3*

Your next patient is a 70-year-old male. 6 months ago, he was bedridden at home for three days due to erysipelas of his right leg. One week later, he developed a DVT with a pulmonary embolism and was brought to the emergency room. Upon arrival, he was unconscious and hemodynamic unstable (blood pressure 80/40 mm Hg, pulse 140/min). He received short-term thrombolytic therapy and unfractionated heparin for 24 hours, after which he recovered hemodynamically. In absence of bleeding complications, apixaban (DOAC) was started.

Before this VTE the patient has had a DVT provoked by immobilization 4 years earlier. In addition, his medical history shows a myocardial infarction 9 years ago, for which acetylsalicylic acid (aspirin, platelet aggregation inhibitor) was started. He smokes and physical examination shows that he is obese (BMI 30) and has a blood pressure of 140/95 mm Hg.

Now 6 months later, he fully recovered from the VTE episode. Today you propose whether to continue apixaban in a reduced dose or not. The acetylsalicylic acid was stopped 6 months ago, when apixaban was started, and will only be restarted if you decide to stop apixaban.

|  | | **5-year risk** |  |
| --- | --- | --- | --- |
| **Without anticoagulation with aspirin** | | |  |
| VTE recurrence | | 7.6% |  |
| Clinically relevant bleeding | | 3.5% |  |
| **With apixaban (reduced) without aspirin** | | |  |
| VTE recurrence | | 2.0% |  |
| Clinically relevant bleeding | | 2.9% |  |
|  | |  | |
| **Guideline** | **Summary recommendation** | | |
| ASH 2020[1] | Suggests stopping anticoagulation | | |
| CHEST 2021[2] | NA | | |
| ESC 2019[3] | Should consider indefinite anticoagulation | | |
| ESVS 2020[4] | NA | | |

*Case vignette 4*

A 27-year-old female presented at the emergency department with chest pain and dyspnea on exertion 4 months ago. She was diagnosed with her first pulmonary embolism for which apixaban (DOAC) was started.

She is overweight (BMI 29) and otherwise healthy. Her father has had multiple VTEs of which she doesn’t know any further details. She has been using a combined oral contraconceptive pill for the past 10 years, including the past 4 months.

Today you propose whether to stop or continue apixaban in a reduced dose. She has no objections to switching to another type of anticonception if necessary.

|  | | **5-year risk** |  |
| --- | --- | --- | --- |
| **Without anticoagulation** | | |  |
| VTE recurrence | | 6.4% |  |
| Clinically relevant bleeding | | 2.0% |  |
| **With apixaban (reduced)** | | |  |
| VTE recurrence | | 1.3% |  |
| Clinically relevant bleeding | | 2.4% |  |
|  | |  | |
| **Guideline** | **Summary recommendation** | | |
| ASH 2020[1] | Typically stopping anticoagulation | | |
| CHEST 2021[2] | Suggests stopping anticoagulation | | |
| ESC 2019[3] | Indefinite anticoagulation should be considered | | |
| ESVS 2020[4] | Indefinite anticoagulation may be considered | | |

*Case vignette 5*

Your patient is a 72-year-old female who presented at the emergency department with dyspnea and coughing caused by an unprovoked pulmonary embolism 3 months ago. A vitamin K antagonist and LMWH were started subsequently. The LMWH could be stopped after 5 days.

Medical history shows a deep vein thrombosis provoked by an ankle fracture and subsequent immobilization of the leg due to a plaster cast 8 years ago. Furthermore, she has type 2 diabetes mellitus and diabetic nephropathy (GFR 25 ml/min/1.73 m^2^). Her blood pressure is 140/90 mm Hg and her haemoglobin level is 7.5 mmol/l (= 12.1 g/dL).

Her INR values have been stable and within target range the past 3 months. Today you propose whether to continue or stop the vitamin K antagonist.

|  | | **5-year risk** |  |
| --- | --- | --- | --- |
| **Without anticoagulation** | | |  |
| VTE recurrence | | 11.0% |  |
| Clinically relevant bleeding | | 3.0% |  |
| **With vitamin K antagonist** | | |  |
| VTE recurrence | | 1.8% |  |
| Clinically relevant bleeding | | 14.9% |  |
|  | |  | |
| **Guideline** | **Summary recommendation** | | |
| ASH 2020[1] | Suggests indefinite anticoagulation | | |
| CHEST 2021[2] | NA | | |
| ESC 2019[3] | Indefinite anticoagulation should be considered | | |
| ESVS 2020[4] | NA | | |

*Case vignette 6*

A 48-year-old male was referred to the emergency department under suspicion of an unprovoked DVT with pulmonary embolism 6 months ago. The diagnosis proximal DVT was confirmed, making it his first VTE, and rivaroxaban (DOAC) was started subsequently. No further diagnostics were performed to establish a pulmonary embolism.

The patient smokes and was recently diagnosed with type 2 diabetes mellitus, for which he uses oral medication. Physical examination shows that he has obesity (BMI 31) and a blood pressure of 150/90 mm Hg.

He has been treated with full dose rivaroxaban for 6 months now. Today you propose whether to continue the rivaroxaban in a reduced dose or stop at all

|  | | **5-year risk** |  |
| --- | --- | --- | --- |
| **Without anticoagulation** | | |  |
| VTE recurrence | | 11.0% |  |
| Clinically relevant bleeding | | 2.3% |  |
| **With vitamin K antagonist** | | |  |
| VTE recurrence | | 1.5% |  |
| Clinically relevant bleeding | | 8.4% |  |
|  | |  | |
| **Guideline** | **Summary recommendation** | | |
| ASH 2020[1] | Suggests indefinite anticoagulation | | |
| CHEST 2021[2] | Recommends indefinite anticoagulation | | |
| ESC 2019[3] | Indefinite anticoagulation should be considered | | |
| ESVS 2020[4] | Recommend indefinite anticoagulation | | |

## Supplementary Text S1.2. Excluded case vignettes.

*Case vignette 7*

A 35-year-old male presented at the emergency department 3 months ago with a red and swollen left leg and mild dyspnea. He was diagnosed with a first unprovoked proximal DVT, presumably with pulmonary embolism, for which apixaban (DOAC) was started.

His medical history comprises only chronic lower back pain for which he daily uses naproxen (NSAID) daily, which he insisted to continue with because other analgesics proved insufficiently effective.

Now, he has been treated with full dose apixaban for 3 months. Today you will propose whether to continue apixaban in a reduced dose or stop at all.

|  | | **5-year risk** |  |
| --- | --- | --- | --- |
| **Without anticoagulation** | | |  |
| VTE recurrence | | 10.8% |  |
| Clinically relevant bleeding | | 2.3% |  |
| **With apixaban (reduced dose)** | | |  |
| VTE recurrence | | 2.2% |  |
| Clinically relevant bleeding | | 2.6% |  |
|  | |  | |
| **Guideline*** | **Summary recommendation** | | |
| ASH 2020[1] | Suggests indefinite anticoagulation | | |
| CHEST 2021[2] | Recommends indefinite anticoagulation | | |
| ESC 2019[3] | Considering indefinite anticoagulation | | |
| ESVS 2020[4] | Recommends indefinite anticoagulation | | |

*Case vignette 8*

An 83-year-old male presented with coughing and chest pain at the emergency department 3 months ago. It appeared to be an unprovoked pulmonary embolism, making it his first VTE, after which rivaroxaban (DOAC) was started.

Medical history shows malignant lymphoma which was diagnosed and treated with chemotherapy 15 years ago and has been in complete remission since, as recently confirmed. The man is overweight (BMI 27) and does not use any relevant medication.

He uses full dose rivaroxaban for 3 months now and today you will propose whether to continue rivaroxaban in a reduced dose or stop it.

|  | | **5-year risk** |  |
| --- | --- | --- | --- |
| **Without anticoagulation** | | |  |
| VTE recurrence | | 16.9% |  |
| Clinically relevant bleeding | | 5.9% |  |
| **With rivaroxaban (reduced)** | | |  |
| VTE recurrence | | 2.3% |  |
| Clinically relevant bleeding | | 20.3% |  |
|  | |  | |
| **Guideline*** | **Summary recommendation** | | |
| ASH 2020[1] | Unclear | | |
| CHEST 2021[2] | Recommends indefinite anticoagulation | | |
| ESC 2019[3] | Considering indefinite anticoagulation | | |
| ESVS 2020[4] | Recommends indefinite anticoagulation | | |

*Case vignette 9*

A 66-year-old man visited the emergency department because of an unprovoked proximal DVT of his right leg 4 months ago. A LMWH and warfarin (VKA) were started simultaneously. The LMWH was stopped after 10 days.

The patient has fatty liver disease and anaemia (7.0 mmol/l = 11.3 g/dL) due to alcohol abuse (6 units/day). He has never had serious bleedings and does not use relevant medicines. Physical examination shows a BMI of 28 and blood pressure of 170/100 mm Hg.

His INR values have been stable and within target range during the past months. Today you propose whether to stop or continue warfarin.

|  | | **5-year risk** |  |
| --- | --- | --- | --- |
| **Without anticoagulation** | | |  |
| VTE recurrence | | 11.0% |  |
| Clinically relevant bleeding | | 3.0% |  |
| **With vitamin K antagonist** | | |  |
| VTE recurrence | | 1.8% |  |
| Clinically relevant bleeding | | 15.0% |  |
|  | |  | |
| **Guideline*** | **Summary recommendation** | | |
| ASH 2020[1] | Suggests indefinite anticoagulation | | |
| CHEST 2021[2] | Recommends indefinite anticoagulation | | |
| ESC 2019[3] | NA | | |
| ESVS 2020[4] | Recommends indefinite anticoagulation | | |

## Supplementary Text S2. Questionnaire on the VTE-PREDICT calculator (Group B)

Q1. Making use of the VTE-PREDICT calculator contributed to my treatment advice regarding continuation/cessation of anticoagulation

Q2. The absolute risks of bleeding and VTE recurrence from the VTE-PREDICT calculator are difficult to translate to clinical practice

Q3. I would find the VTE-PREDICT calculator useful for shared decision making with patients.

Q4. I think the absolute risks derived from the VTE-PREDICT calculator are credible.

Q5. I presently intend to use the VTE-PREDICT calculator regularly

Q6. What did you appreciate most of the VTE-PREDICT calculator?

Q7. Do you have suggestions to improve the VTE-PREDICT calculator?

Q8. Space for other comments on VTE-PREDICT and/or this survey:

**Answer instructions**

Q1-5 must be answered on a 5-point Likert scale (strongly disagree – disagree – neutral – agree – strongly agree). Q6-8 are optional open-end questions. Q1-3 are to investigate usefulness; Q4, credibility; Q5, intention to use.

**References**

1. Davis FD. Perceived Usefulness, Perceived Ease of Use, and User Acceptance of Information Technology. *Source MIS Q* 1989; 13: 319–340.
2. Brooke J. SUS - A quick and dirty usability scale. *Revue des Maladies Respiratoires* 2002; 19: 87–89.
3. Sauro J. SUPR-Q: A Comprehensive Measure of the Quality of the Website User Experience. *J Usability Stud* 2015; 10: 68–86.

## Supplementary Tables S1. Specification of proposed treatment changes within-subject analysis A (no risks provided) vs A (risks provided).

| **Case vignette no.** | | | | | |  |  |  |  |  |
| --- | --- | --- | --- | --- | --- | --- | --- | --- | --- | --- |
| **Change in proposed treatment after using VTE-PREDICT derived data** | **1**  n = 2 | | **2**  n = 26 | **3**  n = 9 | **4**  n = 16 | **5**  n = 21 | **6**  n = 6 | **Overall** n = 81 | |  |
| **Favours shorter OAC duration** |  | |  |  |  |  |  |  | |  |
| Total | 0 (0) | | 18 (69) | 2 (22) | 4 (25) | 14 (67) | 3 (50) | 41 (51) | |  |
| definite 🡪 stop | 0 | | 4 | 0 | 1 | 2 | 1 |  | |  |
| indefinite 🡪 stop | 0 | | 11 | 1 | 2 | 9 | 0 |  | |  |
| indefinite 🡪 definite | 0 | | 3 | 1 | 1 | 3 | 2 |  | |  |
| **Favours extended OAC duration** |  | |  |  |  |  |  |  | |  |
| Total | 1 (50) | | 3 (12) | 5 (56) | 7 (44) | 1 (5) | 0 (0) | 17 (21) | |  |
| stop 🡪 indefinite | 0 | | 0 | 4 | 5 | 0 | 0 |  | |  |
| stop 🡪 definite | 0 | | 1 | 1 | 0 | 0 | 0 |  | |  |
| definite 🡪 indefinite | 1 | | 2 | 0 | 2 | 1 | 0 |  | |  |
| **Other** |  | |  |  |  |  |  |  | |  |
| Total | 1 (50) | | 5 (19) | 2 (22) | 5 (31) | 6 (29) | 3 (50) | 22 (27) | |  |
| stop 🡪 other | 1 | | 0 | 1 | 1 | 0 | 0 |  | |  |
| definite 🡪 other | 0 | | 0 | 0 | 1 | 0 | 1 |  | |  |
| indefinite 🡪 other | 0 | | 0 | 0 | 1 | 3 | 0 |  | |  |
| other 🡪 stop | 0 | | 4 | 0 | 0 | 0 | 0 |  | |  |
| other 🡪 definite | 0 | | 1 | 0 | 1 | 1 | 0 |  | |  |
| other 🡪 indefinite | 0 | | 0 | 1 | 1 | 2 | 2 |  | |  |
| Data is presented as n or n (%).  Stop indicates “stop anticoagulation”; indefinite, “extend anticoagulation indefinitely with periodic re-assessment of risks”; definite, “extend anticoagulation for definite period (e.g. 3 or 9 months)”; other, “I would propose another treatment”.  OAC indicates oral anticoagulation. | | | | | | | | |  |  |

## Supplementary Tables S2. Specification of given answers under response option “I would make another treatment decision:”.

| **Case vignette no.** | | | | | | |  |  |  |
| --- | --- | --- | --- | --- | --- | --- | --- | --- | --- |
| 1 | 2 | | 3 | 4 | 5 | 6 |  |  |  |
| **Group A (no risks provided)** | |  |  | |  |  |  |  |  |
| More information needed | | 0 | 5 | | 0 | 3 | 4 | 1 |  |
| Unclear proposed duration | | 0 | 1 | | 1 | 0 | 0 | 3 |  |
| DOAC instead of VKA | | 0 | NA | | NA | NA | 3 | NA |  |
| **Group A (risks provided)** | |  |  | |  |  |  |  |  |
| More information needed | | 1 | 0 | | 0 | 3 | 1 | 0 |  |
| Unclear proposed duration | | 0 | 0 | | 1 | 1 | 1 | 2 |  |
| DOAC instead of VKA | | 0 | NA | | NA | NA | 3 | NA |  |
| **Group B** | |  |  | |  |  |  |  |  |
| More information needed | | 1 | 1 | | 0 | 4 | 0 | 1 |  |
| Unclear proposed duration | | 0 | 3 | | 1 | 2 | 1 | 2 |  |
| DOAC instead of VKA | | 0 | NA | | NA | NA | 11 | NA |  |
| **Total** | | 2 | 10 | | 3 | 13 | 24 | 9 |  |
| Data is given as number. DOAC indicates direct oral anticoagulant; NA, not applicable; VKA, vitamin K antagonist. | | | | | | | | |  |
|  | | | | | | | | |  |
| **Category** | **Proportion*** | | | **Representative examples of given answers** | | | | | |
| More information needed | 41% | | | 1*. “apply HERDOO2 [risk score] at 6 months”* | | | | | |
|  |  | | | 2. “extended anticoagulation to be *assessed according to REVERSE study”* | | | | | |
| DOAC instead of VKA | 31% | | | 1*. “continue with DOAC indefinitely (reduce dose after 6 months)”* | | | | | |
| Unclear proposed duration | 28% | | | 1*. “reduced dose rivaroxaban”* | | | | | |
| *Among answer option “*I would make another treatment decision*”, chosen a total of 61 times. | | | | | | | | | |
